# Case Report of a New Headache Developed by a Combat Soldier after an Episode of Exertional Heat Illness

**DOI:** 10.3389/fneur.2017.00383

**Published:** 2017-08-07

**Authors:** Haggai Schermann, Mikhail Sherman, Ran Rutenberg

**Affiliations:** ^1^Division of Orthopedic Surgery, Tel Aviv Sourasky Medical Center affiliated with Tel Aviv University, Tel Aviv, Israel; ^2^Heller Institute of Medical Research, Tel Hashomer Sheba Medical Center, Ramat Gan, Israel; ^3^Kirov State Medical University, Kirov, Kirov Oblast, Russia; ^4^Medical Corps, Israeli Defense Forces, Ramat Gan, Israel

**Keywords:** headache, headache disorders, secondary, heat stroke, military personnel, hyperthermia

## Abstract

Only a few authors have reported about a new-onset headache among patients who sustained an episode of an exertional heat illness (EHI). This report presents a healthy and physically fit 20-year-old male who developed a completely new headache after an EHI event. The new headache could be aggravated or called by exertion or exposure to sun and environmental heat. It was severe enough to interfere with even moderate physical activity, but reacted well to a few hours’ rest and OTC pain medications. An extensive work-up including laboratory blood tests, lumbar puncture, head CT, and CT angiogram was negative. The patient remained symptomatic on the 6-month follow-up. Continued abstinence from physical activity and waiting for spontaneous resolution were recommended. We suggest that the headache may be secondary to the hyperthermia brain damage during the EHI event and differs from exertional headache by its association with exposure to sun and environmental heat.

## Introduction

Exertional heat illness (EHI) is a severe life-threatening condition, characterized by an excessive rise of body core temperature and resulting in multi-organ dysfunction (1). Residual neurologic impairment is common and ranges from mild neurobehavioral deficits to quadriplegia (2, 3). Interestingly, only a few authors have reported that survivors of EHI may also develop a new chronic headache. An American physician Silas Mitchell in 1874 described the phenomenon of “novel liability to headache” following either a severe sunstroke, or prolonged and frequent working under direct exposure to sun. Mitchell described having 14 records of patients, whose headache could be triggered or aggravated by exertion, by exposure to sun, and by hot environment (4). More recently, Di Lorenzo et al. reported a case of a 45-year-old woman, who developed new persistent severe daily headaches after a heat stroke (5). HS performed a phone survey of 147 soldiers who had experienced an EHI event between 2008 and 2016 and were referred for work-up and return-to duty decision to a dedicated clinic. About 13 (8.8%) of the soldiers reported having new exercise- and hot weather-related headache that started following the event (unpublished). The patient described here is probably a representative case of what may be defined as post-heat stroke headache.

## Case

Our patient was a healthy Caucasian 20-year-old male sergeant of an Israeli Defense Forces reconnaissance unit. He had a favorable psychosocial background, without personal or family history of headache or neuropsychiatric disorders, no prior head and neck trauma, no chemicals exposure. The patient completed a strenuous 14-month-long special unit training, never having experienced headache or symptoms of heat intolerance. In August 2016, after a 40-km march, he became confused, apathetic, disoriented, walked in zigzag, and could not continue the march. He was subsequently diagnosed with EHI. The patient himself did not recall the event well. The following day, he developed headache and dizziness without vertigo during some trivial activity, was examined by a military physician, and referred to a hospital. At the hospital, general and neurological examination, blood cell count, blood electrolytes, LFTs, CPK, and head CT scan were normal. He consequently resumed routine activity. After about 1 week, he was exposed to bright sunlight and environmental heat for 3 h and again developed severe headache and dizziness. The soldier was transported to a tertiary medical center and underwent a repeated work-up. General and neurological examination, routine blood count tests and LP were negative. Serology for West Nile Virus and Q-fever was negative.

Since the initial work-up, the soldier suffered from headache provoked by sunny and hot weather and moderate exertion, such as running for a distance of 4 km or walking with a backpack for 1 km. He described a frontal headache, with a pressure-like quality, accompanied by mild nausea and photophobia, with frequency of about two or three times a week. The headache would increase gradually until it became severe enough to urge the patient to terminate exertion or seek rest. It ordinarily naturally abated after 3 h or earlier. The headache was partially relieved by a 1 g dipyrone and 500 mg paracetamol with caffeine.

Repeated neurological examination 6 months after the initial event showed normal motor function and sensation, equal reflexes, normal cerebellar tests, negative Romberg test, absent Babinski reflex, and no papilledema on optic examination. CT angiography, ordered to rule out an arteriovenous malformation, was normal. Serology for Q-fever, West Nile Virus was negative. The treating neurologist concluded that the headache is reactive to the episode of hyperthermia and may be expected to improve gradually. The patient was diagnosed by a headache specialty neurologist as having tension-type headache. Given the overall improvement of complaints over time, he was advised a limited duty profile and scheduled for continued follow-up. Abstinence from activity prevented the exertional headache, but he remained symptomatic when exposed to environmental heat, with or without bright sunlight.

## Discussion

The most interesting aspect of our patient’s presentation is temporal relation between EHI and the initiation of complaints, together with the new propensity for headaches upon repeated exposure to sunlight or environmental heat. We asked the patient about the issue of novelty of the complaints several times and carefully reviewed his medical records to confirm the absence of any previous headache and neuropsychiatric complaints. Except for this peculiar feature, the complaints are difficult to discern from primary headache, which commonly presents in this age group and can be influenced by various triggers. However, based on the ICHD-3b criteria, this case cannot be reliably defined as any distinct type of primary headache. The complaints could be classified as “*probable migraine headache*” (1.5.1), having all but one criteria descriptive of classical migraine headache without aura (1.1) (6). The missing criterion is duration of symptoms, which last for only 3 h contrary to 48–72 h required for diagnosis of migraine. Neither does our patient’s clinical picture fit the criteria for tension-type headache. Although the C criteria are in place (bilateral location, pressing quality and mild or moderate intensity), the D criteria (no nausea or vomiting, and no more than one of photophobia or phonophobia) are not satisfied (2.1) (6). Therefore, we consider that the soldier’s complaints may be most appropriately classified among “headache disorders related to disorders of homeostasis” (10.7), or more specifically as a “persistent headache attributed to past disorder of homeostasis” (A10.9) (6). Our case fulfills the criteria of ICHD-3b appendix A10.9 criteria, including the effective treatment of the disorder of homeostasis (B), headache persistence > 3 months (C) and not being better accounted by another ICHD-3 diagnosis. Given the paucity of previous reports of this type of headache, we understand that this diagnosis is debatable and that the main value of this single case is its ability to incite discussion, whether it should be regarded as “probable migraine headache,” or “probable tension-type headache,” or a “heat-related secondary headache.”

The exact pathophysiology of the described phenomenon is not clear. Our patient did not have any occupational exposures associated with development and headache. Military service in a special unit is physically and emotionally demanding and may cause significant distress. However, successful completion of the long training period, being elected for commander role and having no previous psychologist referrals are evidence of effective coping prior to the EHI event. However, psychological stress could possibly play role in development of headache after the initial organic insult, when following strenuous activity the soldier developed exertional hyperthermia and encephalopathy, manifested by confusion. Residual neurologic impairments are reported in medical literature and range from mild neurobehavioral deficits to quadriplegia (2, 3, 7). It has been proposed that in severe heat stroke causes not only hyperthermia itself but also electrolyte disturbances and hypoperfusion due to hemodynamic instability may lead to permanent neurologic damage (3). Di Lorenzo et al. suggested that cytokine fluctuations, caused by the initial hyperthermia, may be involved in the headache mechanism (5). The soldier in this report was hemodynamically stable, but likely had an untreated hyperthermia with spontaneous heat dissipation, which probably lead to acute headache. Our case and the few reports of similar cases in the literature imply that probably more patients with primary headaches may be diagnosed having this particular variant of heat injury-provoked headache. More reports of similar cases would eventually improve our understanding of the characteristic features of this disorder and would enrich the current ICHD-3b A10.9 with additional clinical criteria. The advantage of having a more specific diagnosis may guide further research of its underlying mechanism, prognosis, and treatment options.

## Ethics Statement

Subject presented in this case report had signed a case report consent form.

## Author Contributions

HS has contributed to the design, data acquisition and interpretation, drafted the work, approved the version to be published, and is fully accountable for all aspects of work including its accuracy and integrity. RR contributed to data acquisition, critically reviewed the manuscript, approved its final version, and is accountable for all aspects of the work in ensuring that questions related to the accuracy or integrity of any part of the work are appropriately investigated and resolved. MS contributed by data analysis and interpretation, critical review of the manuscript, approval of the final version and is accountable for all aspects of the work in ensuring that questions related to the accuracy or integrity of any part of the work are appropriately investigated and resolved.

## Conflict of Interest Statement

The authors declare that the research was conducted in the absence of any commercial or financial relationships that could be construed as a potential conflict of interest.
